# Adherence to Mediterranean Diet and Its Association with Metabolic Health Status in Overweight and Obese Adolescents

**DOI:** 10.1155/2022/9925267

**Published:** 2022-08-08

**Authors:** Sobhan Mohammadi, Keyhan Lotfi, Saeideh Mirzaei, Ali Asadi, Masoumeh Akhlaghi, Parvane Saneei

**Affiliations:** ^1^Department of Community Nutrition, School of Nutrition and Food Science, Nutrition and Food Security Research Center, Students' Research Committee, Isfahan University of Medical Sciences, Isfahan, Iran; ^2^Department of Community Nutrition, School of Nutritional Sciences and Dietetics, Tehran University of Medical Sciences, Tehran, Iran; ^3^Department of Community Nutrition, School of Nutrition and Food Science, Shiraz University of Medical Sciences, Shiraz, Iran; ^4^Department of Exercise Physiology, School of Physical Education and Sport Sciences, University of Tehran, Tehran, Iran; ^5^Department of Community, School of Nutrition and Food Science, Nutrition and Food Security Research Center, Isfahan University of Medical Sciences, Isfahan, Iran

## Abstract

**Background:**

Obesity is becoming more prevalent around the world and greatly contributes to chronic disease progression. Previous studies have investigated individual food groups in relation to metabolic health status of adolescents, mainly in Western countries. Limited data are available on the association between dietary patterns and metabolic health in Middle East nations, where childhood overweight/obesity is increasing drastically. Therefore, we investigated the relationship between the Mediterranean diet and metabolic health status among Iranian adolescents.

**Methods:**

This cross-sectional study was conducted on 203 overweight/obese adolescents. Dietary intakes were evaluated by a validated food frequency questionnaire. Anthropometric parameters and blood pressure were measured. Fasting blood samples were obtained to determine circulating insulin, glucose, and lipid profile. Two different methods were applied to classify participants as metabolically healthy obese (MHO) or unhealthy obese (MUO): International Diabetes Federation (IDF) criteria and IDF along with insulin resistance (HOMA-IR) criteria.

**Results:**

A total of 79 (38.9%) and 67 (33.0%) adolescents were, respectively, categorized as MUO, based on IDF and IDF/HOMA definitions. Considering IDF criteria, higher adherence to the Mediterranean diet was related to lower odds of being MUO, both in the crude (OR: 0.17; 95%CI: 0.08–0.37) and fully adjusted model (OR: 0.33; 95% CI: 0.13–0.84). Excluding each component from the score made the association insignificant, except for two components of meat and dairy products. Based on the IDF/HOMA-IR criteria, there was no significant association between Mediterranean diet score and MUO, after considering all potential confounders (OR: 0.47; 95% CI: 0.17–1.30).

**Conclusions:**

We found an inverse association between the Mediterranean diet and odds of MUO among Iranian adolescents, based on IDF criteria. No significant relation was found when MUO was defined based on HOMA-IR/IDF criteria. Further prospective cohort studies are needed to confirm these findings.

## 1. Introduction

In the current century, a great concern has been raised regarding the growing prevalence of overweight and obesity among children and adolescents [1, 2]. Obesity is an important risk factor for metabolic disorders such as type 2 diabetes, dyslipidemia, and hypertension [3]. Also, childhood adiposity is a potential risk factor for being obese at older ages and is related to a higher rate of morbidity and mortality in adulthood [4]. Based on global estimations, the number of children and adolescents with overweight and obesity will be, respectively, 268 and 145 million by 2025 [5]. Approximately 4 million Iranian children and adolescents have been estimated to be overweight and obese by the year 2025 [6]. Along with the excess accumulation of fat in overweight and obese individuals, their metabolic status should be taken into account [7].

Metabolically healthy overweight or obese (MHO) is defined as a situation in which metabolic abnormalities such as elevated fasting blood glucose and blood pressure do not exist concurrently with adiposity [8]. Whereas, higher levels of fasting glucose, triglyceride, and blood pressure do occur in metabolically unhealthy overweight or obese (MUO) individuals [8, 9]. Despite a possible heredity origin for MHO, previous studies have indicated that lifestyle factors might also contribute to the presence of MHO and MUO phenotypes [10]. It is also noteworthy to consider the synergistic effect of dietary intakes and other lifestyle factors on cardiometabolic status [11, 12].

Although higher fruits and vegetables intake has been assumed to be linked to optimal body weight in children, a review study indicated that more evidence is needed to support such an association [13]. Furthermore, a cross-sectional study found no significant difference between intake of specific food groups and MHO/MUO status [14]. However, the nutritional profile of individuals comes from their whole diet, and therefore, taking overall dietary intake into account should be acknowledged. It has been indicated that the MHO phenotype is positively associated with better overall dietary quality [15]. Among dietary patterns, great attention have been recently paid to the Mediterranean diet [16]. The Mediterranean diet recommends high consumption of fruits, vegetables, nuts and legumes, and olive oil; a moderate intake of fish; and low consumption of dairy products as well as red and processed meats [17]. Numerous studies have proposed a significant association between the Mediterranean diet and childhood obesity [18–20]. Furthermore, Lopez et al. reported that higher adherence to the Mediterranean diet has been associated with decreased glucose levels and body mass index in obese children and adolescents [21]. Similarly, another cross-sectional study has suggested that individuals with higher adherence to the Mediterranean dietary pattern were less likely to have an unfavorable metabolic profile [22]. However, the findings of some studies were not in line with these results [23, 24]. Also, considering the high prevalence of overweight and obesity and the subsequent complications among adolescents, investigating the relation of dietary intakes of overweight and obese adolescents with their metabolic health status has considerable importance. As far as we know, the relationship between the Mediterranean dietary pattern and MHO/MUO phenotypes in children and adolescents has received limited attention. Moreover, the majority of earlier studies were done among the European or American populations [22, 25]. Few investigations in this regard have been established in the Middle Eastern region, where dietary intakes are mainly different from Western countries. Therefore, we investigated the association between the Mediterranean diet and healthy or unhealthy phenotypes in Iranian overweight/obese adolescents.

## 2. Methods

### 2.1. Participants

The present cross-sectional study was performed among 203 Iranian adolescents (102 girls and 101 boys) aged 12–18 years. The sample size of the current study was calculated based on the previously published investigations [26, 27], which have shown approximately 60% of overweight and obese adolescents suffered from MUO. Thus, considering a power of 80% and type I error of 0.05, desired confidence interval of 0.95, and precision (d) of 7%, the minimum required sample size was calculated to be 188 subjects. Participants were chosen by the multistage cluster randomly sampled method. Students were selected randomly from sixteen schools in five principal regions of Isfahan, Iran. The weight and height of subjects were measured. Body mass index (BMI) was calculated by dividing weight (kg) by height squared (m^2^) for all students. Then, students were classified as normal weight, overweight, and obese based on the World Health Organization (WHO) age and sex-standardized BMI percentiles [28]. Students who were overweight or obese were invited to participate in the current study. Using this approach, adolescents from a wide range of social and economic statuses were included. Participants who were on a weight-loss diet, those who consume vitamin and mineral supplements, or use medications that might influence body weight, blood glucose, lipid profile, or hypertension were not included. Furthermore, we did not include students with genetic or endocrine problems (such as type 1 diabetes mellitus, hypothyroidism, and Cushing syndrome) in the study. Finally, 203 overweight/obese adolescents were recruited for our study. The written informed agreement was obtained from each participant and his/her parents. The study protocol has been authorized by the Isfahan University of Medical Sciences' Local Ethics Committee.

### 2.2. Dietary Intake Assessment

A validated 147-item food frequency questionnaire (FFQ) was used to collect data on participants' dietary intakes during the preceding year [29]. This FFQ was formerly validated among Iranian adolescents [29]. In addition, previous investigations revealed that this FFQ could reasonably indicate usual dietary intakes and their relations with various disorders among Iranian adolescents [30, 31]. Thus, reasonable validity and reliability were documented for the applied FFQ. A trained nutritionist has completed this FFQ for all participants. Consumption of each food was requested to report based on a daily, weekly, or monthly frequency. Afterward, using household measurements, portion amounts of consumed items were converted to grams per day [32]. The Nutritionist IV software which was based on the USDA food composition database, but was modified for some Iranian foods, was used to calculate nutrients intake.

### 2.3. Assessment of Adherence to the Mediterranean diet

According to the method developed by Trichopoulou et al. [17], we calculated Mediterranean dietary scores (MDS) based on nine components (including vegetables, fruits, legumes, nuts, fish, monounsaturated fatty acids (MUFAs) to saturated fatty acids (SFAs) ratio, grains, meats, and dairy products). Participants received a score of 1 if they were in the top median consumption of vegetables, fruits, legumes, nuts, fish, and MUFAs to SFAs ratio and the bottom median intake of grains, meats, and dairy products. A score of zero was assigned to participants who were in the top median intake of grains, meats and dairy and the bottom median intake of vegetables, fruits, legumes, nuts, fish, and MUFAs to SFAs ratio. The overall Mediterranean diet score for each individual was computed by summing the scores of all nine components. Due to the low consumption of whole grains in Iranian society (10 grams per day), whole and refined grains were combined as a single group of grains in this study [33, 34]. Because of the association between refined grains and several chronic diseases [34, 35], we classified grains as a nonhealthy food group.

### 2.4. Assessment of Anthropometric Indices and Cardio Metabolic Risk Factors

A trained nutritionist used a stadimeter to measure standing height without shoes (to the nearest 0.1 cm). Weight was also measured with a calibrated electronic scale to the nearest 0.1 kg in minimal clothing and without shoes. After normal respiration and without putting any pressure on the body surface, the waist circumference (WC) was measured with a flexible tape (to the nearest 0.1 cm). A Mercury sphygmomanometer with a suitable cuff size was used to measure systolic blood pressure (SBP) and diastolic blood pressure (DBP) on the right arm, twice after a 15-minute recovery time. The average of the two measurements was calculated and used in this study. Blood samples were drawn from all participants after 12 hours of overnight fasting. The concentrations of serum triglycerides (TG), high-density lipoprotein cholesterol (HDL-c), fasting blood glucose (FBG), and insulin were measured. Homeostasis model assessment for insulin resistance (HOMA-IR) was determined using the following formula to measure insulin resistance (IR) [36]: [(fasting insulin (mU/L) × FBG (mmol/L)]/22.5.

### 2.5. Assessment of Metabolic Status

To classify participants as MHO or MUO individuals, two strategies were applied. The first approach was similar to the definition of metabolic syndrome (MetS) based on the children's International Diabetes Federation (IDF) criteria [37]. Being obese without having other MetS components or having only one component of MetS was considered MHO; while MUO children were identified by having two or more MetS components, including increased triglycerides (≥150 mg/dL), decreased HDL-c (<40 mg/dL for the age of <16 y and <50 mg/dL in girls/< 40 mg/dL in boys for the age of ≥16 y), increased fasting blood glucose (≥100 mg/dL), and elevated blood pressure (≥130/85 mmHg). In the second method, HOMA-IR was considered along with IDF criteria that were used in the first classification method. Individuals with HOMA-IR ≥ 3.16 who had at least two metabolic risk factors were classified as MUO and those with HOMA-IR < 3.16 were considered as MHO [9, 38].

### 2.6. Assessment of Other Variables

The Physical Activity Questionnaire for Adolescents (PAQ-A) was applied to assess the participants' physical activity [39]. This questionnaire consists of a 9-item estimating physical activity levels in the last week. The first 8 items asked individuals about (1) spare time activities (i.e., soccer, jogging, swimming, and walking for exercise); their activity during (2) physical education classes, (3) lunch, (4) right after school, (5) evenings, and (6) the last weekend; (7) how they describe their physical activity; and (8) the levels of activity in each day. The first 8 items of the PAQ-A were scored from 1 to 5; a score of 1 indicated the lowest and 5 showed the highest level of physical activity. The average score of all weekdays was considered for the last item. The ninth question evaluated the unusual activity of adolescents during the previous 7 days and was not included in the total score calculation. Finally, participants were classified based on the sum of the scores, as sedentary or not having an orderly week activity (score<2), less active (3<score≤2), active (score≥3), and very active (score≥4). Since a small number of participants were categorized as sedentary and very active, we combined sedentary with less active and active with very active levels to have two final categories (low and high levels of physical activity). A questionnaire was used to record the participants' age, gender, medical history, medication, and supplement use. A validated demographic questionnaire was used by trained researchers to evaluate the socioeconomic status (SES) [40], based on the following variables: parental job, family size, parental education level, having cars in the family, having computers/laptops, having personal room, and taking trips in the year. First, each of these variables was categorized by ordinal scores. Then, these scores were summed up to calculate a total score of SES. Finally, subjects were classified into three levels of SES (low, moderate, and high).

### 2.7. Statistical Analysis

The residual method was used to compute energy-adjusted Mediterranean dietary scores. Then, we classified participants based on energy-adjusted tertiles of MDS. General characteristics of study participants across tertiles of MDS were reported as means ± SDs for continuous and percentages for categorical variables. To examine the differences across tertiles of MDS, we used a one-way analysis of variance (ANOVA) for continuous and chi-square tests for categorical variables. We also used analysis of covariance (ANCOVA) to calculate energy, age, and sex-adjusted dietary intakes of Mediterranean diet components and select nutrients of participants across tertiles of MDS. To identify the association between tertiles of MDS and MUO phenotype, multivariable logistic regression was applied in different models. The odds ratios (OR) and 95% confidence intervals (CI) for MUO status were calculated in crude and adjusted models. In the first model, adjustments were done for age, gender, and energy intake. In the second model, further adjustments for physical activity level and SES were done. In the last model, BMI was added to the previous adjustments. In all models, the first tertile of MDS was considered as the reference category. The odds of having MUO phenotype according to both aforementioned criteria were calculated. Furthermore, we removed each component of MDS at a time from MDS calculation to estimate the effect of that component. Tertiles of MDS were considered as an ordinal variable to examine the trend of odds ratios. SPSS software version 26 (IBM, Chicago, IL) was used for all analyses. *P* values <0.05 (two-sided) were considered as statistically significant.

## 3. Results

### 3.1. General Characteristics of the Study Participants

General characteristics and cardiometabolic factors of the study participants across tertiles of MDS are given in Table 1. Adolescents with the greatest adherence to the Mediterranean diet were more likely to have higher physical activity levels, socioeconomic status levels, and HDL as well as lower systolic blood pressure (mmHg), fasting blood glucose, insulin, HOMA-IR, and triglyceride levels, compared to the lowest adherence (all *P* values<0.05). However, no significant difference was observed in weight, age, BMI, gender, and diastolic blood pressure across tertiles of MDS.

### 3.2. Dietary Intakes of Study Participants

Dietary intakes of study participants according to the tertiles of MDS are given in Table 2. Participants in the top tertile of MDS, compared to those in the bottom tertile, had higher intakes of fruits, vegetables, legumes, nuts, fish, MUFA/SFA, proteins, fats, dietary fibers, omega-3 fatty acids, and magnesium as well as lower consumption of grains, carbohydrates, and vitamin B1 (all *P* values<0.05). No significant differences were observed in energy, meats, and dairy product intake across tertiles of MDS.

**Figure 1:**
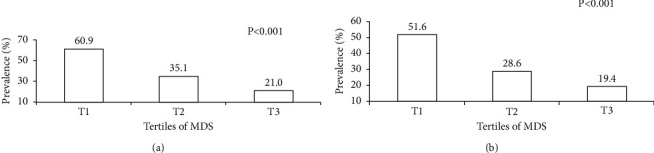
Prevalence of MUO across tertiles of MDS in the study population. (a) Based on IDF definition for MUO. (b) Based on IDF/HOMA-IR definition for MUO.

The prevalence of MUO according to the tertiles of MDS is shown in Figure 1. Based on the IDF criteria, the prevalence of MUO phenotype in the third tertile of MDS was lower than in the first tertile (60.9 vs. 21.0%, *P* < 0.001). A similar result was seen when IDF/HOMA-IR definition was considered (51.6 vs. 19.4%, *P* < 0.001).

### 3.3. ORs for being MUO Across MDS Tertiles

Crude and multivariable-adjusted odds ratios (ORs) for MUO phenotype, based on both definitions, across tertiles of the MDS are given in Table 3. Considering IDF definition, the top tertile of MDS was associated with decreased odds of MUO status in the crude model (OR, 0.17; 95% CI, 0.08–0.37). This association remained significant after including age, gender, and energy intake as confounders in the first model (OR, 0.19; 95% CI, 0.08–0.43). Also, associations were significant after more adjustments for physical activity and SES in the second model (OR, 034; 95% CI, 0.13–0.86), and physical activity, SES, and BMI in the last model (OR, 0.33; 95% CI, 0.13–0.84). For the IDF/HOMA-IR definition, a significant association was observed between the highest tertile of MDS, compared to the lowest one, and the odds of MUO in the crude model (OR, 0.22; 95% CI, 0.10–0.50). Also, after considering age, gender, and energy intake as the covariates, a significant association was observed (OR, 0.26; 95% CI: 0.11–0.61). However, results did not remain significant in the second and third models.  Table 4 provides ORs for being MUO (based on IDF criteria) across MDS, after removing each MDS component at a time, from the total score. In all of the crude models, MDS was associated with a decreased chance of MUO. However, after adjustments for confounding variables, significant associations were only observed after removing meats (OR, 0.39; 95% CI, 0.16–0.92) and dairy products (0.38; 95% CI, 0.16–0.91) from the dietary pattern.  Table 5 provides ORs for being MUO (based on IDF/HOMA-IR criteria) across the MDS, after removing each MDS component at a time, from the total score. MDS was related to lower odds of MUO phenotype in all of the crude models. However, after controlling potential confounders, no significant relation was found.

## 4. Discussion

In the current cross-sectional study, we found that adolescents with the greatest adherence to the Mediterranean diet had lower odds of MUO compared with those with the lowest adherence, based on IDF criteria for MUO phenotype. This association remained significant even after adjustments for potential confounders. In the case of MDS components, excluding each component from the score made the association insignificant, except for two components of meat and dairy products. On the other hand, the association between MDS and MUO, based on IDF/HOMA-IR definition, was dependent on covariates. To the best of our knowledge, this population-based study was the first one examining the Mediterranean diet in relation to MUO phenotype among Iranian adolescents.

According to a longitudinal study, almost half of MHO individuals maintained their metabolic health status in the following years of their lives [41]. It should be highlighted that MHO individuals could develop a metabolically unfavorable phenotype [42]. This condition might lead to a higher risk of morbidity and a reduction in life expectancy [43]. We found an inverse association between the Mediterranean diet and MUO in the current investigation. Hence, it is noteworthy to clinically recommend adolescents to enhance the quality of their diet in order to prevent developing metabolic comorbidities of obesity.

Several previous studies have investigated diet quality in relation to metabolically unhealthy obesity [24, 44]. The association between “priori” dietary scores and metabolic health status in children and adolescents has been adequately addressed [44, 45]. The findings indicated that more adherence to a healthy eating pattern could shift obesity type from MUO to MHO [46]. While the benefits of the Mediterranean diet were widely understood in adults [47], limited data were known regarding this association in children and adolescents. Findings of the current study indicated that greater adherence to the Mediterranean diet might be a protective determinant for MUO, based on IDF definition, among adolescents. However, when we used IDF/HOMA definition for MUO, we found no significant association between MDS and MUO status. Based on the latter definition, adolescents were categorized as MUO/MHO with the consideration of abnormal HOMA-IR in combination with IDF criteria. Some of the MUO cases who were diagnosed based on IDF criteria had normal HOMA-IR and, therefore, were categorized as MHO adolescents in the second definition. Thus, relatively few numbers of cases, which were diagnosed by the IDF/HOMA criteria, could explain the nonsignificant association between MDS and MUO. Similar to our findings, a cross-sectional study among 137 overweight/obese European adolescents found an inverse association between the Mediterranean diet and MUO [22]; the dietary intakes of these adolescents had been assessed by two nonconsecutive 24-hour recalls [22]. Furthermore, in a cross-sectional study on 793 American adolescents with diabetes, FFQ was used to determine dietary intakes [48]. Greater adherence to the Mediterranean diet among these American adolescents was related to improved glycemic control and a preferable lipid profile [48]. On the other hand, a prospective cohort study revealed that a pattern characterized as energy-dense, high in fat, and poor in fiber could be linked to cardiometabolic abnormalities such as elevated insulin levels and insulin resistance [49]. However, according to the findings from a cross-sectional study among 6964 obese American women, dietary intakes were not substantially different between MHO and MUO participants [50]. Also, in a study among Iranian adults, higher adherence to the Mediterranean diet did not indicate a decreased incidence of metabolic syndrome features [51]. The observed discrepancies among findings of previous studies could be clarified by different study designs, study populations, applied questionnaires, and different approaches used in statistical analyses. Furthermore, methods for MDS construction varied among the previous studies. Some studies have considered medians of food groups to define Mediterranean diet scores, while others have used tertiles of intake to construct MDS. Also, differences in the components of the Mediterranean diet might be a reason for these inconsistent findings. Some studies that were conducted in the Mediterranean area included olive oil in the score; whereas, MUFA or the ratio of MUFA to SFA was considered as a component of MDS in non-Mediterranean populations. Therefore, the findings of previous investigations should be cautiously compared.

The cumulative effect of an overall dietary pattern is likely to be stronger than the effect of its individual components [52]. In the current study, after excluding meats and dairy products from MDS, the magnitude of the associations was slightly attenuated, but still remained significant. However, such findings were not observed by exclusion of other components of MDS. We also observed that meats and dairy intakes were not substantially different across tertiles of MDS. Therefore, they might be less important components of the Mediterranean diet than others in relation to MUO. Further studies should be conducted to shed a light on the importance of each component of the Mediterranean diet in relation to health status.

Various underlying mechanisms might explain the observed inverse association between the Mediterranean diet and MUO phenotype, although the exact ones are unknown. The Mediterranean diet has an abundance of plant foods that are rich in antioxidants such as flavonoids, carotenoids, tocopherols, and ascorbic acid [53]. Antioxidants can scavenge free radicals and reduce oxidative stress [53–55]. It is well documented that oxidative stress could be an underlying factor in the development of metabolic disorders [56]. Another probable mechanism is that the Mediterranean diet, characterized by high consumption of vegetables, fruit, and legumes, provides considerable amounts of dietary fiber [57]. Diets rich in fibers could enhance the production of short-chain fatty acids (SCFAs) by gut bacteria [57]. SCFAs could improve glucose and lipid metabolism in many tissues [57]. Furthermore, the Mediterranean diet has a low glycemic index which is related to a lower incidence of insulin resistance [58]. Additionally, adhering to the Mediterranean diet has been inversely related to systemic inflammation [59]. Fish and nuts, as two elements of MDS, are rich sources of omega-3 polyunsaturated fatty acids and could suppress inflammatory processes and enhance endothelial function [60].

The current investigation has some strengths and weaknesses. To our knowledge, this is the first study that examined the association between the Mediterranean diet and MUO among Iranian adolescents. In addition, several potential confounders were taken into account during the analysis. Last, the study was conducted in the Middle East region where available data on diet-disease interaction are limited. However, when interpreting our findings, some limitations must be considered. Our study has a cross-sectional design and we could not infer a causal relationship between the Mediterranean diet and MUO. Therefore, prospective cohort studies are needed to affirm a causal relationship between the Mediterranean diet and MUO. Also, because of using FFQ to evaluate dietary intakes, possible misclassification of subjects was inevitable. Although we assessed BMI and WC to determine obesity and abdominal obesity, we could not evaluate body composition and fat distribution, which could be important in determining metabolic health status. Finally, despite adjustment for several covariates, residual confounders (such as paternal obesity, and sleep disorders) might affect the findings.

In conclusion, we found an inverse relationship between the Mediterranean dietary pattern and odds of MUO among Iranian adolescents based on IDF definition. However, no significant association was found between MDS and MUO phenotypes, considering IDF/HOMA-IR criteria. Greater adherence to the MDS might be beneficial in maintaining metabolic health among overweight/obese adolescents. More prospective studies among different nations are required to confirm these findings.

## Figures and Tables

**Table 1:** General characteristics of study participants across energy-adjusted tertiles of the Mediterranean dietary score (*n* = 203)^1^.

	Tertiles of energy-adjusted MDS^2^			*P* ^3^
	T_1_ (*n* = 64)	T_2_ (*n* = 77)	T_3_ (*n* = 62)	
Age (y)	14.00 ± 1.57	13.99 ± 1.64	13.94 ± 1.63	0.97
Weight (kg)	75.33 ± 12.23	73.97 ± 9.81	70.97 ± 12.69	0.10
Body mass index (kg/m^2^)	27.75 ± 3.40	27.23 ± 1.97	27.10 ± 4.21	0.48
Gender (%)				0.39
Boy	46.9	55.8	45.2	
Girl	53.1	44.2	54.8	
Physical activity levels (%)				<0.001
Low	73.4	46.8	30.6	
High	26.6	53.2	69.4	
Socioeconomic status levels (%)				0.04
Low	39.1	32.5	14.5	
Moderate	40.6	40.3	53.2	
High	20.3	27.3	32.3	
Systolic blood pressure (mmHg)	115.61 ± 12.10	114.44 ± 15.86	107.55 ± 24.72	0.03
Diastolic blood pressure (mmHg)	75.06 ± 10.25	72.99 ± 12.71	72.50 ± 10.72	0.40
Fasting blood glucose (mg/dL)	100.55 ± 9.97	98.40 ± 7.24	95.34 ± 7.60	0.002
Insulin (*µ*UI/mL)	22.77 ± 11.28	21.11 ± 13.65	17.15 ± 12.24	0.04
HOMA-IR index	5.68 ± 3.01	5.17 ± 3.37	4.15 ± 3.29	0.03
Triglycerides (mg/dL)	139.14 ± 66.47	123.10 ± 70.42	102.80 ± 56.95	0.01
HDL cholesterol (mg/dL)	42.95 ± 7.49	44.62 ± 7.94	47.00 ± 7.92	0.02

^1^All values are means ± standard deviation (SD), unless indicated. ^2^Components of MDS adjusted for total energy intake based on the residual method. ^3^Obtained from ANOVA for continuous variables and the chi-square test for categorical variables.

**Table 2:** Multivariable-adjusted intakes of Mediterranean diet components and selected nutrients of study participants across energy-adjusted tertiles of the Mediterranean dietary score (*n* = 203)^1^.

	Tertiles of energy-adjusted MDS^2^			*P* ^3^
	T1 (*n* = 64)	T2 (*n* = 77)	T3 (*n* = 62)	
Energy (Kcal/d)	2904.78 ± 67.79	2926.81 ± 61.99	2806 ± 68.98	0.40

Food groups (g/day):
Fruits	219.90 ± 17.48	343.08 ± 16.00	435.62 ± 17.83	<0.001
Vegetables	190.00 ± 19.98	280.00 ± 18.29	359.99 ± 20.39	<0.001
Meats	68.60 ± 4.09	72.21 ± 3.74	64.39 ± 4.17	0.38
Fish	4.16 ± 0.84	8.42 ± 0.76	10.55 ± 0.85	<0.001
Legumes	31.98 ± 3.19	46.58 ± 2.92	69.29 ± 3.26	<0.001
Nuts	5.85 ± 1.23	12.49 ± 1.13	18.32 ± 1.25	<0.001
Grains	758.31 ± 15.37	651.26 ± 14.07	556.18 ± 15.69	<0.001
Dairy	474.87 ± 27.13	512.65 ± 24.84	561.04 ± 27.69	0.09
MUFA/SFA	0.97 ± 0.02	0.98 ± 0.02	1.10 ± 0.02	<0.001

Other nutrients:
Proteins (% of energy)	13.47 ± 0.24	14.54 ± 0.22	14.89 ± 0.25	<0.001
Fats (% of energy)	27.48 ± 0.64	28.82 ± 0.58	30.29 ± 0.65	0.01
Carbohydrates (% of energy)	60.03 ± 0.63	58.14 ± 0.58	56.70 ± 0.64	0.001
Dietary fiber (g/d)	15.61 ± 0.46	19.26 ± 0.42	23.64 ± 0.47	<0.001
Omega-3 fatty acids (g/d)	0.58 ± 0.02	0.59 ± 0.02	0.65 ± 0.02	0.03
Vitamin B1 (mg/d)	2.77 ± 0.04	2.64 ± 0.03	2.53 ± 0.04	<0.001
Magnesium (mg/d)	246.54 ± 6.86	293.49 ± 6.28	324.58 ± 7.00	<0.001

^1^All values are means ± standard error (SE); energy intake and macronutrients are adjusted for age and gender; all other values are adjusted for age, gender and energy intake. ^2^Components of MDS adjusted for total energy intake based on the residual method. ^3^Obtained from ANCOVA.

**Table 3:** Multivariable-adjusted odds ratio for MUO across energy-adjusted tertiles of the Mediterranean dietary score (*n* = 203)^1^.

	Tertiles of energy-adjusted MDS^2^			*P*_*tren* *d*_
	T1 (*n* = 64)	T2 (*n* = 77)	T3 (*n* = 62)	
MUO based on IDF criteria				
MUO cases (*n*)	39	27	13	
Crude	1.00	0.35 (0.17–0.69)	0.17 (0.08–0.37)	<0.001
Model 1	1.00	0.32 (0.16–0.65)	0.19 (0.08–0.43)	<0.001
Model 2	1.00	0.43 (0.19–0.94)	0.34 (0.13–0.86)	0.01
Model 3	1.00	0.43 (0.20–0.96)	0.33 (0.13–0.84)	0.01

MUO based on IDF/HOMA-IR criteria				
MUO cases (n)	33	22	12	
Crude	1.00	0.38 (0.19–0.75)	0.22 (0.10–0.50)	<0.001
Model 1	1.00	0.32 (0.15–0.67)	0.26 (0.11–0.61)	<0.001
Model 2	1.00	0.44 (0.19–0.99)	0.50 (0.19–1.32)	0.09
Model 3	1.00	0.46 (0.20–1.03)	0.47 (0.17–1.30)	0.08

^1^All values are odds ratios and 95% confidence intervals. P_trend_ was obtained by the use of tertiles of MDS as an ordinal variable in the model. Model 1, adjusted for age, gender, energy intake. Model 2, more adjustments for physical activity levels and socioeconomic status. Model 3, further adjustment for BMI. ^2^Components of MDS adjusted for total energy intake based on the residual method.

**Table 4:** Multivariable-adjusted odds ratio for MUO (based on IDF criteria) across energy-adjusted tertiles of the Mediterranean dietary score after excluding each component from the score (*n* = 203)^1^.

	Tertiles of energy-adjusted MDS^3^			*P*_*tren* *d*_
	T1 (*n* = 64)	T2 (*n* = 77)	T3 (*n* = 62)	
MDS minus vegetables				
Crude	1.00	0.51 (0.23–1.12)	0.38 (0.20–0.73)	0.003
Multivariable-adjusted^2^	1.00	0.49 (0.19–1.25)	0.55 (0.25–1.20)	0.11

MDS minus fruits				
Crude	1.00	0.60 (0.29–1.22)	0.29 (0.14–0.59)	0.001
Multivariable-adjusted^2^	1.00	0.79 (0.34–1.82)	0.47 (0.20–1.09)	0.08

MDS minus legumes				
Crude	1.00	0.35 (0.16–0.75)	0.30 (0.15–0.59)	<0.001
Multivariable-adjusted^2^	1.00	0.64 (0.26–1.57)	0.46 (0.21–1.04)	0.06

MDS minus nuts				
Crude	1.00	0.70 (0.34–1.47)	0.28 (0.14–0.57)	<0.001
Multivariable-adjusted^2^	1.00	0.80 (0.33–1.92)	0.45 (0.20–1.04)	0.07

MDS minus MUFA : SFA ratio				
Crude	1.00	0.53 (0.25–1.13)	0.27 (0.14–0.54)	<0.001
Multivariable-adjusted^2^	1.00	0.71 (0.29–1.73)	0.47 (0.21–1.06)	0.07

MDS minus fish				
Crude	1.00	0.52 (0.25–1.10)	0.30 (0.15–0.59)	<0.001
Multivariable-adjusted^2^	1.00	0.57 (0.24–1.35)	0.51 (0.22–1.16)	0.08

MDS minus grains				
Crude	1.00	0.63 (0.30–1.34)	0.29 (0.15–0.57)	<0.001
Multivariable-adjusted^2^	1.00	0.72 (0.30–1.75)	0.48 (0.21–1.09)	0.08

MDS minus meats				
Crude	1.00	0.24 (0.11–0.52)	0.21 (0.11–0.43)	<0.001
Multivariable-adjusted^2^	1.00	0.20 (0.08–0.49)	0.39 (0.16–0.92)	0.09

MDS minus dairy				
Crude	1.00	0.26 (0.12–0.55)	0.18 (0.08–0.38)	<0.001
Multivariable-adjusted^2^	1.00	0.38 (0.16–0.89)	0.38 (0.16–0.91)	0.03

^1^All values are odds ratios and 95% confidence intervals. P_trend_ was obtained by the use of tertiles of MDS as an ordinal variable in the model. ^2^Adjusted for age, gender, energy intake, physical activity levels, socioeconomic status and BMI. ^3^Components of MDS adjusted for total energy intake based on the residual method.

**Table 5:** Multivariable-adjusted odds ratio for MUO (based on IDF/HOMA-IR criteria) across energy-adjusted tertiles of the Mediterranean dietary score after excluding each component from the score (*n* = 203)^1^.

	Tertiles of energy-adjusted MDS^3^			*P*_*tren* *d*_
	T1 (*n* = 64)	T2 (*n* = 77)	T3 (*n* = 62)	
MDS minus vegetables				
Crude	1.00	0.42 (0.18–0.99)	0.50 (.0.26–0.96)	0.03
Multivariable-adjusted^2^	1.00	0.39 (0.14–1.05)	0.80 (0.35–1.83)	0.44

MDS minus fruits				
Crude	1.00	0.60 (0.29–1.25)	0.38 (0.19–0.78)	0.01
Multivariable-adjusted^2^	1.00	0.76 (0.32–1.82)	0.66 (0.27–1.62)	0.34

MDS minus legumes				
Crude	1.00	0.40 (0.18–0.90)	0.38 (0.19–0.76)	0.005
Multivariable-adjusted^2^	1.00	0.80 (0.31–2.05)	0.64 (0.27–1.52)	0.31

MDS minus nuts				
Crude	1.00	0.62 (0.29–1.33)	0.31 (0.15–0.65)	0.002
Multivariable-adjusted^2^	1.00	0.65 (0.26–1.63)	0.52 (0.21–1.28)	0.13

MDS minus MUFA : SFA ratio				
Crude	1.00	0.64 (0.29–1.39)	0.35 (0.17–0.70)	0.003
Multivariable-adjusted^2^	1.00	0.85 (0.34–2.13)	0.66 (0.28–1.55)	0.34

MDS minus fish				
Crude	1.00	0.62 (0.29–1.33)	0.37 (0.18–0.75)	0.005
Multivariable-adjusted^2^	1.00	0.74 (0.30–1.81)	0.68 (0.28–1.65)	0.36

MDS minus grains				
Crude	1.00	0.65 (0.30–1.40)	0.35 (0.17–0.70)	0.003
Multivariable-adjusted^2^	1.00	0.65 (0.26–1.63)	0.61 (0.26–1.44)	0.23

MDS minus meats				
Crude	1.00	0.27 (0.12–0.61)	0.24 (0.12–0.49)	<0.001
Multivariable-adjusted^2^	1.00	0.22 (0.09–0.57)	0.44 (0.17–1.12)	0.02

MDS minus dairy				
Crude	1.00	0.31 (0.15–0.67)	0.25 (0.12–0.53)	<0.001
Multivariable-adjusted^2^	1.00	0.45 (0.19–1.06)	0.57 (0.23–1.42)	0.19

^1^All values are odds ratios and 95% confidence intervals. P_trend_ was obtained by the use of tertiles of MDS as an ordinal variable in the model. ^2^Adjusted for age, gender, energy intake, physical activity levels, socioeconomic status and BMI. ^3^Components of MDS adjusted for total energy intake based on the residual method.

## Data Availability

The data used to support the findings of this study are available from the corresponding author upon request.

## Abbreviations:

FFQ: Food frequency questionnaire
OR: Odds ratios
95% CI: 95% confidence interval
BMI: Body mass index
MHO: Metabolically healthy obesity
MUO: Metabolically unhealthy obesity
IDF: International Diabetes Federation
HOMA-IR: Homeostasis Model Assessment for Insulin Resistance
PAQ-A: Physical Activity Questionnaire for Adolescents
SES: Socioeconomic status
MDS: Mediterranean diet score
WHO: World Health Organization
USDA: United States Department of Agriculture
MUFA: Mono unsaturated fatty acids
SFA: Saturated fatty acids
SCFA: Short-chain fatty acids
WC: Waist circumference
SBP: Systolic blood pressure
DBP: Diastolic blood pressure
TG: Triglycerides
HDL-c: High-density lipoprotein cholesterol
FBG: Fasting blood glucose
ANOVA: Analysis of variance
ANCOVA: Analysis of covariance
SPSS: Statistical Package for Social Sciences.
